# Understanding large-scale, long-term larval connectivity patterns: The case of the Northern Line Islands in the Central Pacific Ocean

**DOI:** 10.1371/journal.pone.0182681

**Published:** 2017-08-15

**Authors:** Lorenzo Mari, Luca Bonaventura, Andrea Storto, Paco Melià, Marino Gatto, Simona Masina, Renato Casagrandi

**Affiliations:** 1 Dipartimento di Elettronica, Informazione e Bioingegneria, Politecnico di Milano, 20133 Milano, Italy; 2 MOX, Dipartimento di Matematica, Politecnico di Milano, Italy; 3 Fondazione Centro Euro-Mediterraneo sui Cambiamenti Climatici (CMCC), Bologna, Italy; 4 Istituto Nazionale di Geofisica e Vulcanologia (INGV), Bologna, Italy; University of Padova, ITALY

## Abstract

Protecting key hotspots of marine biodiversity is essential to maintain ecosystem services at large spatial scales. Protected areas serve not only as sources of propagules colonizing other habitats, but also as receptors, thus acting as protected nurseries. To quantify the geographical extent and the temporal persistence of ecological benefits resulting from protection, we investigate larval connectivity within a remote archipelago, characterized by a strong spatial gradient of human impact from pristine to heavily exploited: the Northern Line Islands (NLIs), including part of the Pacific Remote Islands Marine National Monument (PRI-MNM). Larvae are described as passive Lagrangian particles transported by oceanic currents obtained from a oceanographic reanalysis. We compare different simulation schemes and compute connectivity measures (larval exchange probabilities and minimum/average larval dispersal distances from target islands). To explore the role of PRI-MNM in protecting marine organisms with pelagic larval stages, we drive millions of individual-based simulations for various Pelagic Larval Durations (PLDs), in all release seasons, and over a two-decades time horizon (1991–2010). We find that connectivity in the NLIs is spatially asymmetric and displays significant intra- and inter-annual variations. The islands belonging to PRI-MNM act more as sinks than sources of larvae, and connectivity is higher during the winter-spring period. In multi-annual analyses, yearly averaged southward connectivity significantly and negatively correlates with climatological anomalies (El Niño). This points out a possible system fragility and susceptibility to global warming. Quantitative assessments of large-scale, long-term marine connectivity patterns help understand region-specific, ecologically relevant interactions between islands. This is fundamental for devising scientifically-based protection strategies, which must be space- and time-varying to cope with the challenges posed by the concurrent pressures of human exploitation and global climate change.

## Introduction

Multiple anthropogenic pressures are causing dramatic ecological changes to the oceans at the global scale [1]. Preservation of the marine environment requires the creation and the protection of sanctuaries, as well as the conservation and, if necessary, the restoration of the most critical seascape ecological functions [2]. Connectivity is a vital element of landscape and seascape structure [3], resulting from the interaction between the physical environment and the movement of organisms among different areas and habitats. Connectivity determines the amount of between-patch dispersal, thus being one of the most important mechanisms ensuring the preservation of marine biodiversity [4]. Indeed, ecological connectivity is a key concept for a variety of conservation and management issues in marine environments. These include securing the persistence and long-term viability of species assemblages [5], enhancing ecosystem resilience (i.e. facilitating recovery after disturbance, see [6]), and improving productivity and ecosystem functioning in the sea [7].

**Fig 1:**
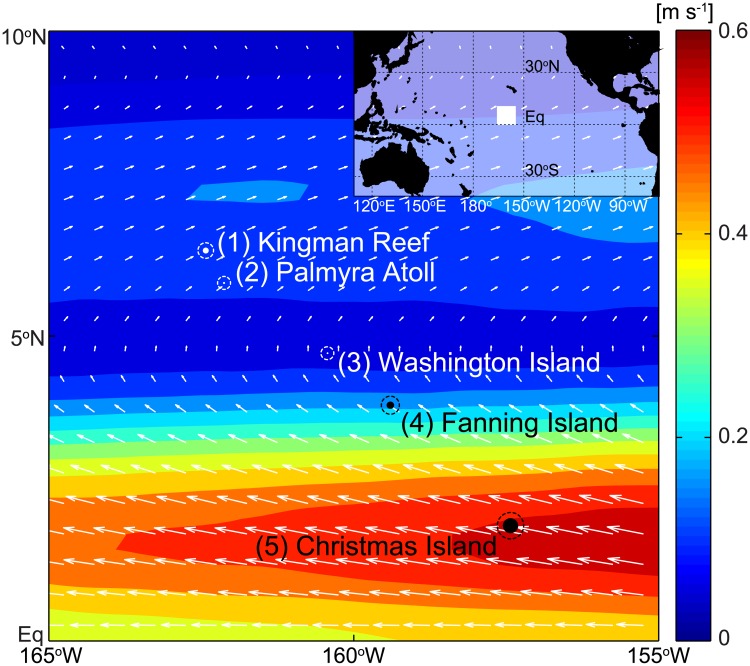
Study area. Filled dots indicate positions and areal extents of the five considered islands of the Northern Line Islands archipelago, from where larvae are released for Lagrangian simulations, while dashed circles enclose the area used as retention zone of each island (see Methods). Colors represent the magnitude of the surface velocity field (global average 1991–2010), while arrows indicate average flow direction. The map is highlighted as a white square in the Central Pacific region (inset).

It is thus a matter of the uttermost importance to assess connectivity in marine regions where there exist species that risk extinction, but might be rescued by means of Marine Protected Areas (MPAs). These can serve as refuges for adult organisms, nurseries for juveniles and sources of propagules for other unprotected areas. A paradigmatic example of this situation is that of the Northern Line Islands (NLIs) archipelago, which is located in the Central Pacific Ocean (Fig 1). It is one of the longest island chains of the planet and is characterized by a strong spatial gradient of human impact. In fact, the northernmost islands, Palmyra Atoll and Kingman Reef (territories of the United States) are uninhabited and are among the most pristine tropical marine environments worldwide [8]. Both are part of the Pacific Remote Islands Marine National Monument, the largest marine reserve in the world, which has been extended to 1.3 million km^2^ by a proclamation of the US Presidency in 2014. The southern part of the NLIs, instead, belongs to the Republic of Kiribati and consists of inhabited islands, whose residents heavily depend on fishing for their subsistence.

Here we use Lagrangian simulations to explore the connectivity patterns that link the protected islands in the North to the impacted islands in the South, thus providing flows of propagules that may sustain commercial fishing. Lagrangian simulations have already proved to be powerful tools to effectively explore connectivity in marine environments, mainly at single-species level (but see [9], and references therein, for metacommunity approaches). Two considerations concur in encouraging the use of Lagrangian simulations for the quantification of marine connectivity. On one hand, oceanographers have put a lot of effort into the development of eddy-permitting Ocean Global Circulation Models (OGCMs) that can also assimilate both remotely-sensed and in situ data [10]. The reliability of these models in reproducing oceanic currents at increasingly finer spatial (in the order of few kilometers), and temporal (daily) resolutions is steadily increasing over time [11]. On the other hand, the major dispersal events during the lifetime of many marine species occur at the beginning of their life, when individuals are larvae or small propagules (natal dispersal). Although compelling evidence exists that larvae are capable of active motion and navigation (*sensu* [12]; see e.g. [13, 14, 15, 16, 17]), it may still be plausible to assume that larval dispersal at large spatial scales is mostly driven by oceanic currents.

Therefore, to quantitatively estimate the ecological between-island connectivity, we run simulations of organisms’ movements assuming that larvae are Lagrangian particles dispersing in accordance with oceanic currents. Of course, the spatial scale of larval movements is much smaller than the typical resolution of OGCMs. It is therefore necessary to suitably downscale the oceanic circulation fields obtained from global models. To this end we investigate which among the most commonly used interpolation methods is the most appropriate, thus assessing the trade-off between quality of results and computational cost. Then, we determine connectivity by using different indicators based on the probability of successful larval exchange and the distribution of minimum distances of released larvae from a target island.

We discuss how and to what extent the timing (season) of the organisms’ release and the duration of their dispersal period (Pelagic Larval Duration, PLD) influence the spatio-temporal measures of connectivity between source and sink areas. In addition to intra-annual variations, we explore inter-annual changes by performing our analyses over time windows as long as 20 years (1991–2010). We conclude our study discussing the robustness of the identified connectivity patterns and analyzing the possible link of the connectivity indicators with climate variations.

## Materials and methods

### Study area

The study area is represented by the Line Islands, a chain of eleven atolls (eight of which are part of the Republic of Kiribati, the others are grouped with the United States Minor Outlying Islands, US) and low coral islands in the central Pacific Ocean, south of the Hawaiian Islands. The archipelago stretches over 2,350 km in a northwest-southeast direction, thus being one of the longest island chains of the world. The archipelago is geographically divided into three subgroups: Northern, Central, and Southern Line Islands. Here we specifically focus on the five most important Northern Line Islands (NLIs, Fig 1), which represent an ideal setting to analyze connectivity patterns. In fact, despite their similar oceanographic characteristics (they are all located in the Intertropical Convergence Zone, an area influenced by the equatorial countercurrent), the recent history of the NLIs is characterized by an anthropogenic trajectory from virtually unimpacted (e.g. Kingman Reef and Palmyra Atoll in the north) to heavily impacted (e.g. Christmas Island-Kiritimati), following a gradient of fishing pressure and human impact increasing from north to south [8].

The oceanographic environment of the archipelago is influenced by strong zonal and meridional gradients in temperature and precipitation, and characterized by sea surface temperatures above 27°C and intense zonal currents with sharp direction change. From the dynamical viewpoint (see again Fig 1), the southernmost island (Christmas) is completely embedded in the South Equatorial Current (SEC), a strong zonal current directed from East to West in the same direction of the trade winds and reaching velocities up to 1 m/s. Moving northward, near Fanning Island, the prevailing current starts to switch direction, and the region is characterized by high variability from intra-seasonal to interannual time scales. At approximately 5°N the zonal currents become very weak because the SEC starts to be replaced by the North Equatorial Counter Current (NECC) flowing from west to east. The dominant prevailing current near the two northernmost islands is the NECC. This region, embedded within the Intertropical Convergence Zone, is characterized by high precipitation. Overall, the equatorial Pacific region is the center of the strongest interannual variability of the global climate system.

### Oceanic model

The hydrodynamic engine for the individual-based simulations of larval dispersal described below is the Ocean General Circulation Model (OGCM) NEMO (version 3.2, [18]), coupled with the Louvain-La-Neuve sea-ice dynamic and thermodynamic model (LIM2, [19]). The model has a resolution of approximately 1/4 of degree, ranging from about 10 km at high latitudes to 22 km at mid-latitudes and 27 km at the Equator. The grid is tripolar [20], with two poles on the Asian and North-American continents and the third on the South Pole. The model has 50 vertical depth levels with partial steps [21]. The OGCM implements a free surface formulation [22]. At the surface boundary, the ocean is forced by the ERA-Interim atmospheric reanalysis produced by the European Center for Medium Range Weather Forecast [23] through the use of the CORE bulk formulas [24], with the total precipitation corrected according to [25]. The model also takes advantage of a surface nudging scheme to relax sea-surface temperature (SST), salinity (SSS) and sea-ice concentration to observed values, provided by the NOAA SST daily analyses [26], the EN3 objective analyses [27] and the NASA sea-ice analysis [28], respectively. In particular, SST and SSS are relaxed with a time-scale of 12 and 300 days, respectively, to correct the biases of the heat and freshwater fluxes coming from the atmospheric forcing. The OGCM simulation spans the period 1979-2011 and has been initialized from the ocean at rest, with initial conditions for temperature and salinity provided by the World Ocean Atlas 2005 climatology [29]. After a spin-up period of two years, the model output is provided as daily means, thus allowing a high-frequency update of the hydrodynamic fields.

### Simulated larval dispersal

Dispersal patterns are investigated via individual-based simulations, in which fish larvae are described as passive Lagrangian particles (e.g. [30, 31, 32]) released at each of the five NLIs and then transported by oceanic currents. In each numerical experiment *n* = 20,000 particles are released from each island. The initial coordinates of the particles are randomly generated within round patches whose positions and sizes match those of the NLIs, while the initial depth of each particle is drawn from a uniform distribution with support in the 0–50 m depth interval. Lagrangian particles are released on the first day of a given month and subsequently tracked for a timespan corresponding to their pelagic larval duration (PLD). Particle tracking is performed by integrating the motion equations of each particle via the standard Euler explicit scheme, with a time step of 3 hours. Metric distances are used to compute particle net displacement (*sensu* [33]) at each time step. The vertical component of the velocity field is disregarded, because it is at least one order of magnitude smaller than the horizontal components of the velocity field produced by the circulation model. Therefore, a particle released at any initial depth will remain at the same depth throughout the simulation. Particles are tracked until the end of their PLD or until they eventually exit the spatial domain embedding the NLIs (165°–155°W, 0°–10°N; see again see Fig 1).

Arguably, a higher-resolution circulation model would allow a more detailed description of larval dispersal and, in turn, a more reliable estimation of between-atoll connectivity. In particular, high-resolution circulation fields would especially be useful to describe the initial and the final phases of the trajectories describing larval transport dynamics (propagules escaping from inner lagoons at source atolls and seeking for reef sites at destination atolls). Ideally, a multi-scale approach (see e.g. [16]) coupling high-resolution fields around the atolls with a relatively coarser description of oceanic circulation would likely represent a gold standard in terms of balancing accuracy and parsimony. However, since we aim to describe connectivity both at a large spatial scale (hundreds of kilometers) and on a long temporal window (two decades) using realistic circulation fields (i.e. as produced by an oceanic reanalysis), the OGCM NEMO represents a valuable tool, even if its resolution cannot accommodate a complete description of all the nouances of between-atoll dispersal.

Month of release and PLD represent two typical life traits of fish species, reflecting different reproduction schedules and strategies (e.g. broadcast spawners with long PLDs vs. brooders with short PLDs). Here, they are assumed to be independent of each other and of any other environmental variable (such as water temperature and pH). Coupled with the variability of oceanic currents, spawning season and PLD are expected to have a remarkable impact on larval dispersal patterns and between-island connectivity. Therefore, we explore wide ranges of these two parameters (for PLD: from one week to four months; for spawning season: 12 release dates from January 1st to December 1st) and perform an exhaustive sensitivity analysis to estimate their effects on large-scale connectivity patterns. On the other hand, we do not attempt here to provide Lagrangian particles with any of the biological or behavioral characteristics that can be included in more detailed, species-specific studies (see e.g. [9, 16, 34]). In particular, we refrain from introducing any features pertaining to active larval movement (swimming, orientation, navigation, vertical migration, reef-seeking behavior) or further details about the pelagic phase of the dispersing organisms (competency, mortality) so as to perform a simulation exercise that is as general and species-*non*specific as possible. We note that general patterns could also be sought by means of a comprehensive numerical exploration of suitable ranges of the parameters describing larval behavior and biology; however, given the complexity of the problem (see e.g. [14, 16, 17, 35, 36]), this alternative approach would make any exhaustive simulation strategy computationally unaffordable.

### Comparison of interpolation schemes

Although the simulations occur along bidimensional surfaces, the evaluation of Lagrangian trajectories requires three-dimensional (3D) spatial interpolation of the circulation fields. Therefore, before engaging in Lagrangian simulations, several interpolation schemes are benchmarked to assess possible differences in their performances for the problem at hand. Specifically (for details, see [37]), a linear interpolant for scattered data using Delaunay tessellation (T), a linear nearest-neighbor scheme for gridded data (L) and a cubic spline interpolant for gridded data (S) are employed (S1 Fig). A regular latitude/longitude grid corresponding to the outputs of the circulation model is used with algorithms L and S, while the corresponding irregular metric grid is used for scheme T. The performances of the different interpolants are evaluated via a non-exhaustive cross-validation procedure in which a grid point is randomly selected and the relevant longitudinal/latitudinal velocity components discarded from the circulation field; current velocity in the selected grid point is then estimated via interpolation (with each of the three numerical schemes), and absolute/relative reconstruction errors are computed; the procedure is repeated many times for different grid/time points (10,000 for each year from 1991 to 2010). Subgrid interpolation exercises are also performed, in which the outcomes of the different algorithms are evaluated at grid mid-points. Finally, more demanding interpolation experiments are also run, in which the whole velocity field is reconstructed based on thinned subsets obtained by removing 3D data strips (rows/columns/layers for latitude/longitude/depth) of variable width *s* (*s* = [1, 2, 3, 4]).

### Connectivity measures

Lagrangian simulations are used to derive connectivity patterns between the five NLIs. Note that we actually study *potential* connectivity, because neither larval behavior/biology (see above) nor the population dynamics of adult fish (including e.g. demographic feedback on propagule production) are considered. In a Lagrangian framework, the connectivity between two islands *i* and *j* is determined by successful dispersal events from *i* to *j*, i.e. by particles released at island *i* approaching island *j* during their PLD. Therefore, the minimum (great-circle) distance $D_{ij}^{k}$ to island *j* of a trajectory *k* starting from island *i* is an important indicator of potential dispersal success for a single larva. As *n* Lagrangian particles are released at each island, the ensemble minimum $D_{ij}^{\text{min}}$ and average $D_{ij}^{\text{ave}}$ of the individual trajectories’ minimum distances $D_{ij}^{k}$ are two useful measures of potential connectivity. The former suggests in fact whether between-island connectivity is possible at all: large $D_{ij}^{\text{min}}$ indicates that no particles released at island *i* can approach island *j*, even in the most favourable cases. The second, instead, is indicative of the intensity of between-island connections: small $D_{ij}^{\text{ave}}$’s indicate that a significant share of the particles released at *i* can be found (at some time during their PLD) in the proximity of *j*. Although here we limit our attention to these two statistical indicators, others (such as the percentiles of the minimum distance distributions) could be usefully evaluated as well.

Based on individual trajectories, connectivity between islands *i* and *j* is hereafter measured as the fraction *P*_*ij*_ of particles released at *i* whose trajectories cross (at any time before the end of the PLD being considered) a suitably defined retention region of island *j* (e.g. [38]). As soon as a trajectory starting at island *i* crosses one of the retention regions of any island *j* ≠ *i* the simulation is stopped and the dispersal event is considered successful. More sophisticated mechanisms to determine whether a particle has successfully reached a landing site could indeed be designed, yet at the expense of adding some speculations on top of an already relatively complex set-up. Self-connectivity is produced by particles that either remain confined within the retention region of their originating island for the whole PLD or that cross it again after having left it.

The retention region of each island is centered at the island coordinates and is simply described as a round patch with radius corresponding to that of the release region plus a buffer *β*. Introducing buffered retention regions is required in our approach because i) the spatial resolution of the oceanographic simulations is not high enough to resolve accurately the effects of each island on the circulation fields, and ii) no larval reef-seeking behavior is accounted for in our Lagrangian simulations, as already mentioned above. Note that this choice is relatively common in the literature, with values of *β* spanning from 1 km to tens of kilometers (e.g. [31, 38, 39, 40, 41]). For consistency with the horizontal resolution of the oceanographic reanalyses, here we explore the range 0 ≤ *β* ≤ 20 km (*β* = 10 km in Fig 1). With our definition of retention region, it is possible to derive the potential connectivity network (*sensu* [42]) between the different islands of the archipelago.

### Long-term analyses

Lagrangian simulations and the evaluation or potential connectivity measures are performed over a 20-year-long timespan (namely from 1991 to 2010), as not only intra- but also inter-annual variability of the circulation fields can play a major role in determining connectivity patterns (and the ensuing ecological dynamics; see [43, 44]). Also, we look for connections with the most important climatic driver acting in the region, namely ENSO (region Niño 3.4; data available at http://www.cpc.ncep.noaa.gov/data/indices/). The significance of such connections is evaluated through linear regression analysis, performed via standard least square techniques (e.g. [45]). Palmyra Atoll, which hosts some of the best preserved reefs in the NLIs archipelago and thus represents an important biodiversity conservation hotspot (see e.g. [46]), is used as focal island for the analysis of connectivity patterns.

## Results

### Choice of interpolation algorithm

**Fig 2:**
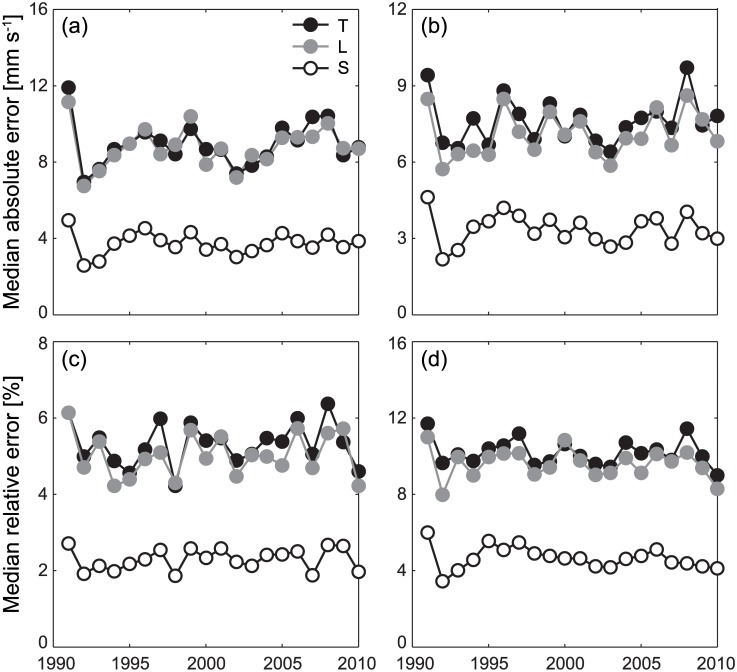
Evaluation of different interpolation algorithms for sub-grid reconstruction of the oceanic velocity fields. (a–b) Median absolute reconstruction errors for the longitudinal (a) and the latitudinal (b) components of the velocity fields. (c–d) As in a–b, for the relative reconstruction errors. See Methods for details on the cross-validation procedure used to evaluate interpolation errors and S1 Fig for a schematic representation of the different interpolation schemes.

Cross-validation shows that the performances of the three interpolation algorithms are quite different, with scheme S (spline interpolant) consistently outperforming schemes T and L (linear schemes; see Fig 2). Despite some small fluctuations in recostruction performances induced by the interannual variability of the oceanic currents, the yearly median values of the relative errors produced by scheme S are always around 2%/4% for the longitudinal/latitudinal components of the velocity field, while median relative errors are around 5%/10% for schemes T and L. As for subgrid interpolation tests, the predictions of the three schemes at grid mid-points lie within a median range of about 3 mm s^−1^ for both longitudinal and latitudinal components, corresponding to a median relative range of about 2%/4% of the velocity values estimated with scheme S. These figures, reported here for reference, have been obtained with the velocity fields of year 2000, yet they are representative of the performances of the interpolation schemes for all the other years as well (not shown). Reconstruction of whole velocity fields from thinned subsets shows some variability in the three algorithms’ performances as well. The best reconstruction performances with moderately thinned datasets are obtained with scheme S, while scheme L may be better suited for heavily thinned datasets (S2 Fig). From all these tests, we conclude that scheme S represents the best interpolation tool for the problem at hand, in which spatial interpolation of the velocity fields for individual-based simulations of larval movement is always performed at sub-grid scale. The spline interpolant is thus retained as the reference interpolation algorithm for all the subsequent numerical analyses. Results obtained with schemes T and L are reported as Supporting Figs.

### Potential connectivity via Lagrangian simulations

**Fig 3:**
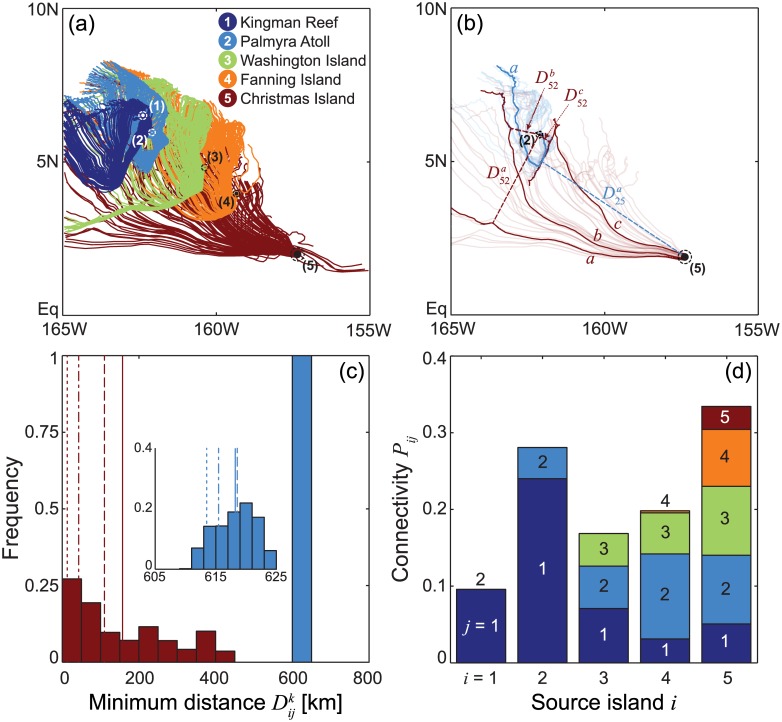
Simulation of particle dispersal with a PLD of 2 months and release on March 1st, 2000. (a) Sample trajectories of Lagrangian particles released from each of the NLIs; filled circles indicate positions and areal extents of the islands, while dashed circles mark their retention zones. (b) Analysis of sample trajectories for Lagrangian particles released from Palmyra Atoll (2, sky blue) and Christmas Island (5, burgundy); first, the minimum distance from each of the NLIs is computed for all trajectories, then ensemble statistics are evaluated. (c) Frequency distribution (50 km bins) of the minimum distances to Palmyra Atoll of the trajectories starting at Christmas Island ($D_{52}^{k}$, burgundy), and viceversa ($D_{25}^{k}$, sky blue; inset shows the latter distribution at a finer spatial grain); solid, dashed, dotted and dash-dotted lines mark respectively the positions of the average, median, 10th and 25th percentiles of the frequency distributions (for $D_{52}^{k}$: 157, 110, 13, 43 km; for $D_{25}^{k}$: 618, 619, 613, 615 km). (d) Histogram visualization of the between-island connectivity matrix; bars represent the fraction of particles released at source island *i* (horizontal axis) that cross the retention zone of island *j* (*β* = 10 kilometers, island numbers and colors as in panel a).

The circulation fields from 1991–2010 are used to drive Lagrangian simulations of larval dispersal. Fig 3 shows the results of a numerical experiment in which larvae/particles are released on March 1st, 2000, and tracked for a PLD of two months. The individual trajectories (panel a) indicate that currents predominantly flow from South-East to North-West during the considered timespan, thus favoring a remarkable connectivity along that direction. Because of the intrinsic anisotropy of ocean currents, between-island connectivity is expected to be highly heterogeneous. Specifically, in the experiment shown in Fig 3, the southernmost islands turn out to be potential sources for propagule dispersal, while the northernmost islands could better be described as sinks. These features clearly emerge from the analysis of the particles’ trajectories, specifically from the evaluation of the minimum distances $D_{ij}^{k}$ and their distributions (panels b and c). As an example, a sizable fraction of the particles released from Christmas Island may eventually approach Palmyra Atoll (more than 25% with minimum distance < 50 km), while particles released from Palmyra Atoll never approach Christmas Island (all trajectories with minimum distance > 600 km), thus obviously precluding any ecologically relevant connectivity. These results can be used to build the connectivity matrix displayed as a histogram in panel d, which has been evaluated with retention regions defined by a buffer *β* of 10 km. According to the Lagrangian simulation performed in the temporal window of Fig 3, almost 10% of the particles leaving from Kingman Reef are retained locally, while very few of them can reach Palmyra Atoll (and none the other islands of the archipelago). Conversely, about 25% of the particles released at Palmyra Atoll reach Kingman Reef. Quite interestingly, Kingman Reef and Palmyra are expected to receive significant amounts of particles (3–11%) also from Washington, Fanning and Christmas Islands, which are located about 300, 450 and 750 km away from Palmyra, respectively. In this numerical experiment self-connectivity is found to be significant (3–10%) for all islands but Fanning. Although Lagrangian trajectories produced by the three interpolation schemes do not differ much upon visual inspection (contrast Fig 3 with S3 Fig), some quantitative differences in between-island connectivity scores do emerge, especially regarding self-retention, or the connectivity between Kingman Reef and Palmyra Atoll.

### Spawning seasons, PLDs and connectivity patterns

**Fig 4:**
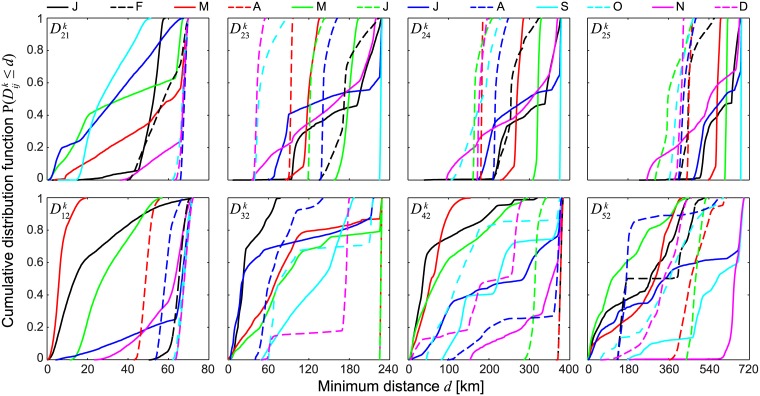
Minimum distances to target islands of particles starting from (or ending to) Palmyra Atoll. Cumulative distributions of the minimum distances to/from the other NLIs (numbered as in Fig 1) for trajectories having Palmyra Atoll as a source/sink (top/bottom panels). Results are shown for a PLD of 60 days and different release months (see legend), and refer to year 2000.

The robustness and generality of the results obtained for a specific month (Fig 3) need to be tested in the context of the natural variability of circulation fields. Fig 4 shows that the seasonal changes of the currents in one year strongly influence the distributions of the distances $D_{ij}^{k}$, with remarkable differences even for trajectories starting during months that are relatively close to each other. The analysis of the cumulative distance distributions from/to Palmyra Atoll also shows that connectivity between some islands pairs is *de facto* prevented for all release seasons because of the geography of the currents and the spatial arrangement of the NLIs, at least for the PLD and the temporal frame considered in Fig 4 (60 days, year 2000). This is the case, for instance, for connectivity from Palmyra Atoll to the three southernmost islands of the archipelago. Conversely, the distance distributions are such that connectivity to Palmyra Atoll from all of the other NLIs is likely to be guaranteed, at least during some specific months.

**Fig 5:**
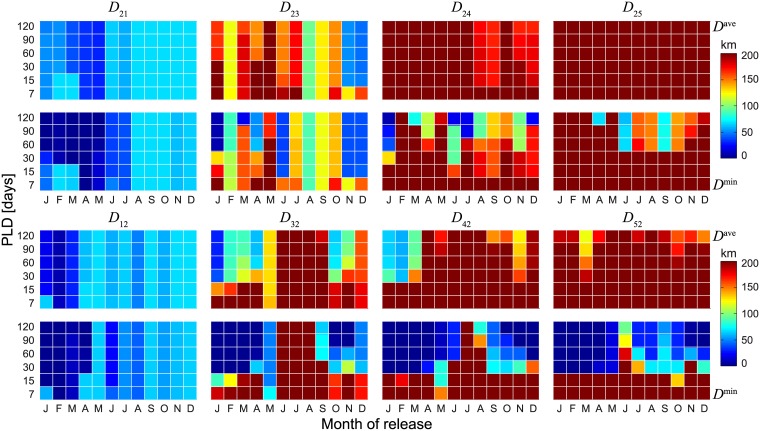
Effects of PLDs on distances to target islands of particles starting from (or ending to) Palmyra Atoll. Ensemble minimum and average distances of individual Lagrangian trajectories from (*D*_2*i*_, top) or to (*D*_*i*2_, bottom) Palmyra Atoll, as a function of release season and PLD. Results refer to year 2000. Spatial interpolation (for Lagrangian tracking) has been performed via algorithm S (spline interpolation).

Ensemble indicators of how trajectories change in response to different PLD/spawning season scenarios are reported in Fig 5 for year 2000. As somehow expected, in general both average and minimum distances decrease with increasing PLDs, but they appear to be quite variable. Similarly to what has been shown in Fig 4, no simple indications can be drawn about the relation between average/minimum distances and the release season, although patterns linked to the seasonal variability of ocean currents clearly emerge. Distance distributions and ensemble indicators are consistently estimated by Lagrangian simulations run with different interpolation algorithms (contrast Figs 4 and 5, obtained with scheme S, with Figs S4 and S5 Figs obtained with scheme T, or Figs S6 and S7 Figs obtained with scheme L). However, some noticeable differences can be found for trajectories starting from Fanning and Christmas Islands, i.e. the islands that are located furthest from Palmyra Atoll.

**Fig 6:**
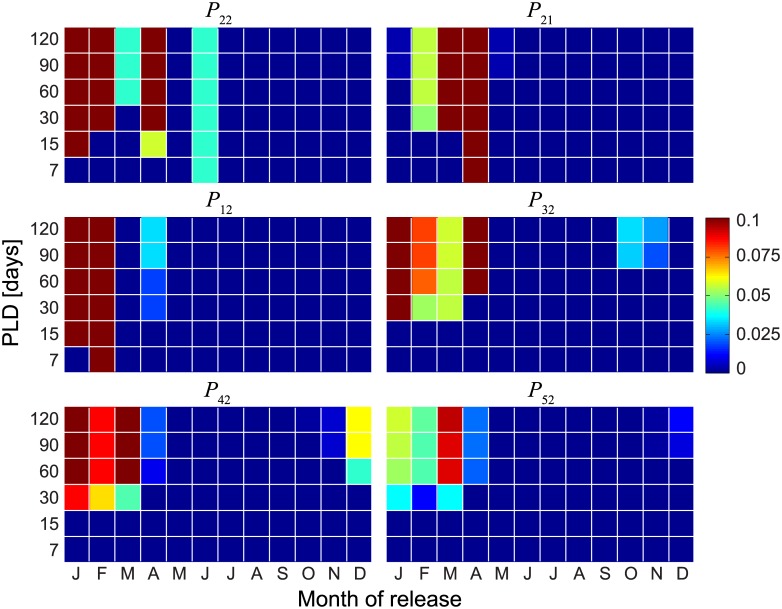
Connectivities for Palmyra Atoll. Self- (*P*_22_), out- (*P*_2*j*_) and in-bound (*P*_*i*2_) connectivity for Palmyra Atoll as a function of spawning season and PLD. Results refer to year 2000 and are obtained for *β* = 10 km. Connectivity patterns to Washington, Fanning and Christmas Islands are not shown because connectivity scores are less than 1% for every combination of PLD and release season.

**Fig 7:**
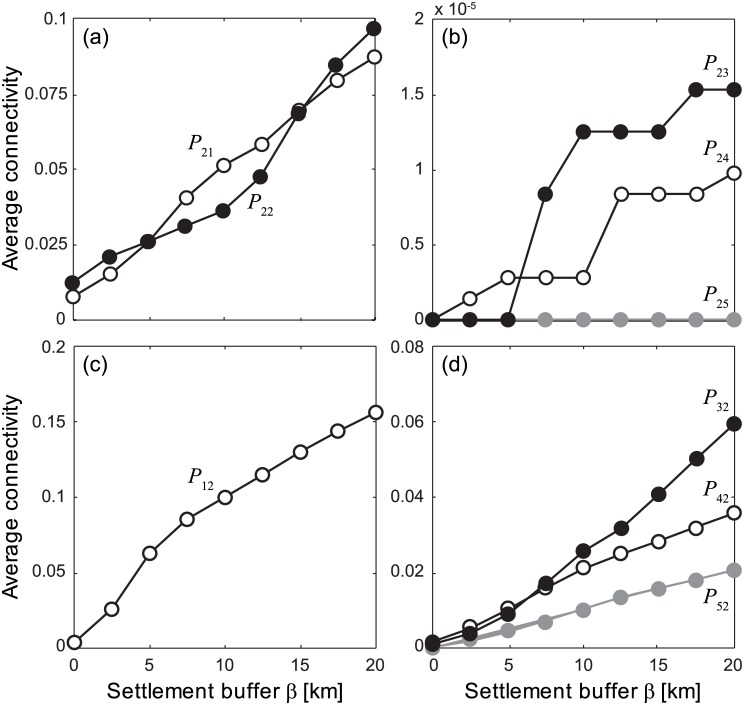
The effect of settlement buffer on connectivity. Connectivity scores of Palmyra Atoll for different sizes of the settlement buffer (*β*) that defines the retention region of each island. Connectivity patterns are evaluated from Lagrangian simulations run with Palmyra Atoll as a source (panels a–b) or a sink (panels c–d), using the interpolation scheme S. Results refer to year 2000, and have been averaged over different PLDs and release seasons.

PLDs and spawning seasons influence potential connectivity patterns too, as shown in Fig 6, which reports connectivity scores from (*P*_2*i*_) and to (*P*_*i*2_) Palmyra Atoll (for year 2000, as in Figs 4–5, and buffer size *β* = 10 km). Longer PLDs are found to favor connectivity, as expected from the definition given in the Methods section. Larval retention is present for the months from January to June (except for May), with potential connectivity scores sometimes exceeding 10%. Palmyra Atoll appears to act as a potential propagule source for Kingman Reef (from January to May, but mostly in March and April), while no outbound connectivity to the other islands is observed. As a sink, Palmyra Atoll receives propagules from all the other islands of the archipelago. Connectivity to Palmyra Atoll is especially strong for Lagrangian trajectories originated during January—April. Some connectivity is also observed from the three southernmost islands between October and December. Results are not qualitatively altered by the choice of the interpolant for Lagrangian tracking (contrast Fig 6, obtained with scheme S, with Figs S8 and S9 Figs, obtained with schemes T and L, respectively), but some quantitative differences emerge also in this case. Connectivity pattern do obviously depend on the size of the retention zone of each island, i.e. on buffer size *β*. A sensitivity analysis of the connectivity scores relevant to Palmyra Atoll (averaged over different PLDs and release seasons, year 2000) with respect to variations of the buffer size (Fig 7) shows that potential connectivity expectedly increases when considering larger values of *β*. Interestingly, the average connectivity scores evaluated from Lagrangian simulations run with different interpolation algorithms show a remarkable quantitative agreement (S10 Fig), with the largest absolute differences among the three interpolants being around 1% (e.g. for connectivity from Washington Island to Palmyra Atoll, *P*_32_, with 10 < *β* < 20 km). It is worth that the average connectivities within the PRI-MNM (first column panels of Fig 7) reach quite significant values, order of magnitudes higher than the north-to-south connections from Palmyra Atoll to the other human inhabited NLIs and well above to the ecologically relevant south-to-north connectivities from them to Palmyra (second column panels of Fig 7).

### Multi-annual connectivity patterns

**Fig 8:**
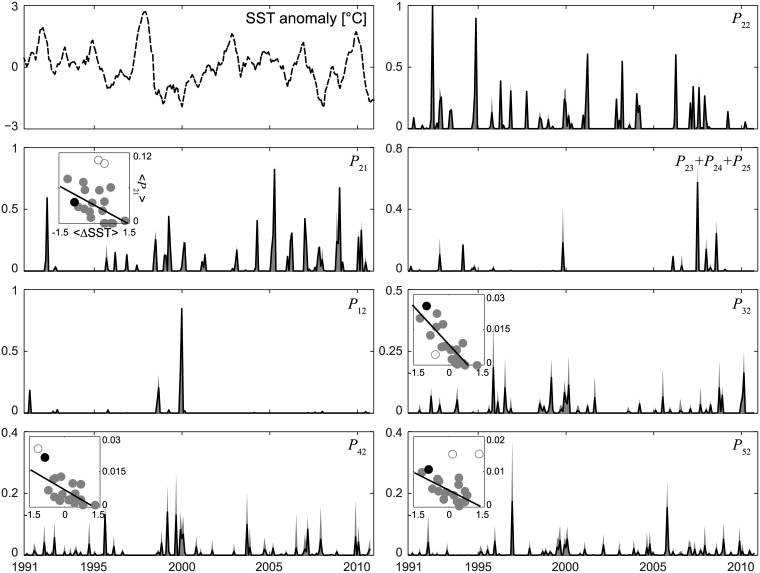
Relationship between ENSO SST anomaly and connectivity. ENSO SST anomaly (top-left panel) and connectivity patterns for the period 1991–2010. Black solid lines represent monthly connectivity scores (obtained with *β* = 10 km) averaged over the set of PLDs considered in Fig 6, while the gray shaded areas report the min-max range of the connectivity scores. Insets are reported only for statistically significant correlations (*p* < 0.05) between yearly averages of the SST anomaly (〈*ΔSST*〉) and yearly averages of connectivity scores (〈*P*_*ij*_〉, dots; year 2000 is highlighted in black). Regression lines obtained after the removal of outliers (white filled dots), identified by computing confidence intervals for each observation based on the associated (externally) studentized residuals (see e.g. [45, 47]), are shown as solid lines within insets.

All the results reported in Figs 4–7 refer to year 2000, yet potential connectivity might significantly fluctuate from year to year, following interannual variations of the circulation fields. Fig 8 illustrates how connectivity patterns between the five NLIs have changed over a twenty-year timespan, namely from 1991 to 2010 (connectivity scores averaged over PLDs, *β* = 10 km). This long-term analysis shows that remarkable interannual variations do actually exist. At the same time, the connectivity scores reported in Fig 6 appear to be far from extraordinary (possibly except for connectivity *P*_12_ from Kingman Reef to Palmyra Atoll, which actually peaked in year 2000, i.e. the year analyzed in Fig 6). Average connectivity patterns are pretty robust to the choice of interpolation algorithm, as it can be verified by contrasting Fig 8 (obtained from Lagrangian simulations run with a spline interpolant, scheme S), with Figs S11 and S12 Figs (obtained with linear schemes T and L, respectively).

As connectivity patterns appear to be fairly erratic, it is difficult to identify any clearly visible connection between the monthly ENSO anomaly in the climatic region 3.4 (top-left panel in Fig 8) and the average monthly connectivity patterns from/to Palmyra Atoll. It is worthwhile to remind that an anomaly exceeding 0.5°C signals the appearance of an El Niño event, while an anomaly lower than -0.5°C corresponds to La Niña. Interestingly, some statistically significant (*p* < 0.05) correlations do actually emerge from our analysis. Specifically, yearly averages of the south-to-north connectivity scores (i.e. from Palmyra Atoll to Kingman Reef and from the three southernmost islands to Palmyra) are negatively correlated with the yearly averages of the ENSO signal (i.e. the higher the sea surface temperature anomaly, the lower the connectivity). No other connectivity pattern involving Palmyra as either source or sink shows any statistically significant trend. Remarkably, multi-annual statistical trends are relatively robust to changes in buffer size *β*. Table 1 shows in fact that all the statistically significant correlations between connectivity scores and SST anomalies are negative. In particular, the average connectivities from the southernmost islands to Palmyra Atoll are (all but *P*_52_ evaluated for *β* = 5 km) negatively correlated with the ENSO signal independently of the values of *β* considered here, while a statistically significative correlation between ENSO and Palmyra-to-Kingman connectivity is found for *β* > 5 km only. The correlation analysis presented in Table 1 refers to connectivity patterns evaluated from Lagrangian simulations run with scheme S for spatial interpolation. Results obtained with the other interpolants (see figures S11 and S12 Figs) are very similar, although the results obtained with a linear interpolation scheme show significant negative correlations between ENSO and Palmyra-to-Kingman connectivity for either small (*β* ≤ 5) or large (*β* > 10) retention regions.

**Table 1:** Correlation between connectivity scores (*P*_2*_ = *P*_23_ + *P*_24_ + *P*_25_) and ENSO anomaly (yearly averages) for different buffer sizes (*β* in kilometres). Shown are Pearson coefficients for statistically significant correlations only (*p* < 0.05). All statistically significant correlations are negative (see text).

	*β* = 0	2.5	5	7.5	10	12.5	15	17.5	20
*P*_22_	-	-	-	-	-	-	-	-	-
*P*_21_	-	-	-	-0.48	-0.55	-0.59	-0.59	-0.60	-0.56
*P*_2*_	-	-	-	-	-	-	-	-	-
*P*_12_	-	-	-	-	-	-	-	-	-
*P*_32_	-0.60	-0.72	-0.86	-0.89	-0.84	-0.86	-0.86	-0.78	-0.75
*P*_42_	-0.70	-0.70	-0.71	-0.72	-0.63	-0.62	-0.59	-0.56	-0.53
*P*_52_	-0.72	-0.65	-	-0.62	-0.64	-0.65	-0.66	-0.65	-0.64

## Discussion

**Fig 9:**
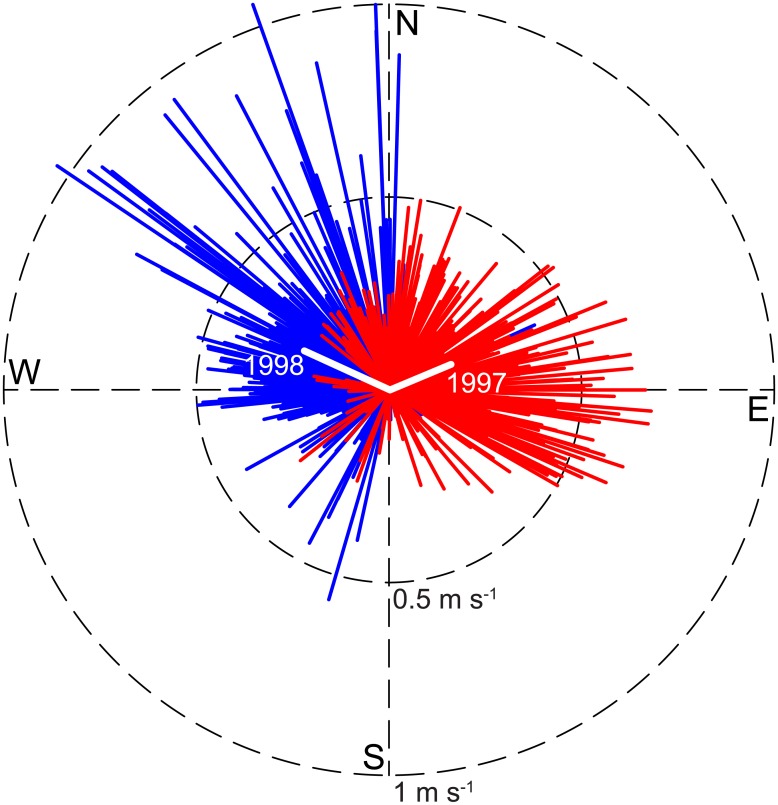
Circulation fields and ENSO anomalies. Daily- and yearly-averaged (colored and white, respectively) directions and intensities of the local circulation field for a representative location in the study area (160°W, 5°N, close to Washington Island), for two different years (1997, red, and 1998, blue). Year 1997 was characterized by a strong El Niño anomaly, while 1998 marked the beginning of a protracted La Niña event, with remarkable reverberations on superficial ocean currents.

In this work we have assessed larval connectivity patterns in the NLIs (Central Pacific Ocean) by performing Lagrangian simulations based on currents retrieved from the OGCM NEMO over time scal“es ranging from one month to twenty years (1991–2010). Although the model has quite a fine spatial resolution (considering that it has been developed as a global-scale reanalysis), some interpolation algorithm is needed for obtaining motion fields in every possible position reached by larvae within the relevant domain. Spline interpolation schemes provide better results in terms of the simulated trajectories and their geometrical properties compared to linear interpolants. Overall, though, the obtained connectivity patterns seem to be pretty robust with respect to the interpolation scheme. The same robustness holds for long-term analyses. In particular, our results suggest a clear and significant pattern of negative correlation between ENSO and the potential between-island connectivity scores. The dynamical and thermodynamical conditions of the NLIs are influenced by the phase of the El Niño Southern Oscillation (ENSO), the dominant air-sea coupled mode of natural variability of the region [48]. The explanation of this association has to be sought in the striking differences observed in ocean currents during different ENSO phases. As a notable, exemplificative comparison, Fig 9 shows (in a sort of “El Niño/La Niña rose”) the directions and intensities of the daily- and yearly-averaged local circulation fields nearby Washington island during El Niño vs. La Niña years. They revealed to be almost orthogonal in this case: the otherwise dominant South-East to North-West circulation is in fact deeply altered during El-Niño, a factor which explains the reduction of connectivity in years of positive 〈Δ*SST*〉, as shown in Fig 8.

In addition to a standard index of connectivity, i.e. the probability that larvae departing from a source island will reach a sink island, we have introduced indices based on distances travelled by moving larvae and analyzed their distributions. Interesting patterns do emerge, of both island- and season-dependent nature. Our attention has been focused, in particular, on the twin islands of the Pacific Remote Islands Marine National Monument, Palmyra Atoll and Kingman Reef, because of their undoubtable ecological value not only at the local but also the regional scale. Despite the temporal intermittency of some features—like retention at Palmyra Atoll, for example—the signals of connectivity are quite clear in terms of directions and intensities. At all the temporal scales here analyzed, these two northernmost islands act more as sinks of particles from the southernmost part of the archipelago than as sources of larvae for it. A closer look at these two key hotspots of biodiversity reveals that, while Palmyra Atoll plays an important, yet intermittent, role as a potential source of larvae towards Kingman Reef, the opposite is not true (except for some exceptional years). However, when we consider Palmyra and Kingman jointly, the retention within the island complex is very high, which guarantees that the sanctuary might possibly go on functioning even without the import of larvae from south. Nevertheless, the protected areas extend their conservation power well beyond their borders. In fact, even the larvae released by the more impacted islands can ultimately reach the sanctuary, which may therefore serve as a nursery for juveniles originally born elsewhere. Conversely, larval dispersal from Palmyra to the southern islands seems to be sporadic, thus unlikely to be ecologically important. It is worth remarking that the increasing anthropogenic pressures in action on Washington, Fanning and Christmas islands might turn the South-to-North connectivity into a risk rather than an opportunity. In fact, any pollutants or alien species possibly departing from the southern islands, which are the most inhabited by humans, have non-negligible chances of reaching Palmyra Atoll and/or Kingman Reef.

Models for assessing ecological connectivity within the seascape, especially if based on oceanographic simulations, may help not only to design conservation policies, but also to test their robustness with respect to ongoing global changes. In particular, the IPCC 5th report on climate change (WG1, The Physical Basis, [49]) predicts steadily increasing SST under any scenario and strongest ocean warming for the surface in tropical regions. Also, AR5 has confirmed that “there is *high confidence* that the ENSO will remain the dominant mode of interannual variability in the tropical Pacific, with global effects in the 21st century”. Therefore, the present study’s result on the negative correlation between ENSO and the connectivity within the NLIs between 1991-2010 is potentially of great importance. It points out that global warming has not only direct effects on marine organisms’ physiology and autoecology, but can also adversely influence the marine ecosystems’ functioning, thus rendering them quite fragile and vulnerable. This calls for an ever greater attention to the large-scale management of human pressures on our oceans.

## Supporting information

S1 Fig The three interpolation schemes. Schematic representation of the three interpolation schemes used in this work applied to a hypothetical bivariate quadratic function. (a) Linear interpolant for scattered data using Delaunay triangulation (scheme T in the main text). (b) Linear interpolant for gridded data (scheme L). (c) Spline interpolant for gridded data (scheme S). 3D versions of the algorithms have been used for the interpolation of velocity fields in Lagrangian simulations.
(EPS)

S2 Fig Reconstruction of velocity fields averaged over year 2000 from thinned subsets (insets of panel d). (a–b) Median absolute reconstruction errors for the longitudinal (a) and latitudinal (b) components of the velocity fields. (c–d) As in a–b, for the relative reconstruction errors. The inset of panel d portrays a thinned dataset for *s* = 1, with black dots indicating data points used for field reconstruction. See Methods for details on data thinning, choice of interpolants (color-coded as in panel a) and field reconstruction.
(EPS)

S3 Fig Simulation of particle dispersal. (a–c) As in panels a, c and d of Fig 3, but spatial interpolation for Lagrangian tracking has been performed with scheme T. (d–f) As in panels a–c, with scheme L. See text for details on Lagrangian simulations and the related interpolation issues.
(EPS)

S4 Fig Minimum distances of particles with the T-interpolation scheme. Cumulative distributions of the minimum distances to/from the other NLIs for trajectories having Palmyra Atoll as a source/sink (top/bottom panels). Details as in Fig 4, except for the choice of the interpolant (here scheme T is used instead of scheme S).
(EPS)

S5 Fig Minimum distances of particles with the L-interpolation scheme. Cumulative distributions of the minimum distances to/from the other NLIs for trajectories having Palmyra Atoll as a source/sink (top/bottom panels). Details as in Fig 4, except for the choice of the interpolant (here scheme L is used instead of scheme S).
(EPS)

S6 Fig Ensemble minimum and average distances with the T-interpolation scheme. Ensemble minimum and average distances of individual Lagrangian trajectories from (top) or to (bottom) Palmyra Atoll. Details as in Fig 5, except for the choice of the interpolant (here scheme T is used instead of scheme S).
(EPS)

S7 Fig Ensemble minimum and average distances with the L-interpolation scheme. Ensemble minimum and average distances of individual Lagrangian trajectories from (top) or to (bottom) Palmyra Atoll. Details as in Fig 5, except for the choice of the interpolant (here scheme L is used instead of scheme S).
(EPS)

S8 Fig Connectivity for Palmyra Atoll with the T-interpolation scheme. Self- (*P*_22_), out- (*P*_2*j*_) and in-bound (*P*_*i*2_) connectivity for Palmyra Atoll. Details as in Fig 6, except for the choice of the interpolant (here scheme T is used instead of scheme S).
(EPS)

S9 Fig Connectivity for Palmyra Atoll with the L-interpolation scheme. Self- (*P*_22_), out- (*P*_2*j*_) and in-bound (*P*_*i*2_) connectivity for Palmyra Atoll. Details as in Fig 6, except for the choice of the interpolant (here scheme L is used instead of scheme S).
(EPS)

S10 Fig The effect of settlement buffer on connectivity. Connectivity scores for different sizes of the settlement buffer (*β*) that defines the retention region of each island. Connectivity patterns are evaluated from Lagrangian simulations run with different interpolation schemes (see legend in the top-left panel). Results refer to year 2000, and have been averaged over different PLDs and release seasons.
(EPS)

S11 Fig Effects of different interpolation schemes on the relationship between ENSO-SST anomaly and potential connectivity in the NLIs: The T-interpolation scheme. ENSO-SST anomaly (top-left panel) and monthly connectivity patterns for the period 1991–2010. Details as in Fig 8, except for the choice of the interpolant (here scheme T is used instead of scheme S).
(EPS)

S12 Fig Effects of different interpolation schemes on the relationship between ENSO-SST anomaly and potential connectivity in the NLIs: The L-interpolation scheme. ENSO-SST anomaly (top-left panel) and monthly connectivity patterns for the period 1991–2010. Details as in Fig 8, except for the choice of the interpolant (here scheme L is used instead of scheme S).
(EPS)
